# Utility of post mastectomy radiotherapy among patients with T1/ T2 N1 disease: A retrospective cohort study

**DOI:** 10.1016/j.amsu.2021.102295

**Published:** 2021-04-16

**Authors:** Lubna M. Vohra, Rufina Soomro, Dua Jabeen, Nasir Ali, Nargis Khan

**Affiliations:** a Department of Breast Surgery, Aga Khan University Hospital, Karachi, Pakistan; b Department of Surgery, Liaquat National Hospital and Medical College, Karachi, Pakistan; c Jinnah Sindh Medical University, Karachi, Pakistan; d Department of Radiation Oncology, Aga Khan University Hospital, Karachi, Pakistan; e Department of Surgery, Aga Khan University Hospital, Karachi, Pakistan

**Keywords:** Mastectomy, Radiotherapy, Lymph nodes, Breast cancer, Recurrence, Survival, Patients

## Abstract

**Background:**

Pakistan has the highest incidence of breast cancer among Asian Countries but there is insufficient representation of local data addressing breast cancer treatment and outcome. We sought to determine the role of post-mastectomy radiotherapy (PMRT) in T1- T2 breast cancer with 1–3 positive axillary lymph nodes.

**Methods:**

Data was reviewed retrospectively of total 755 patients out of which 291 received PMRT and 464 did not from two large breast cancer centres.

**Results:**

With a median follow up of 78 months, 4 (4.5%) patients developed loco regional recurrence (LRR) in the PMRT group while a substantial number 74 (24.4%) recurred in the non PMRT group (p = 0.000). Loco regional free survival rate (LRFS) and overall survival rate (OS) was significantly better for PMRT patients than non-PMRT patients (P = <0.000). Multivariate analysis identified young age, lymphovascular invasion, extra capsular extension, triple negative and ER/PR negative were independent prognostic factors affecting loco regional free survival (LRFS).

**Conclusion:**

Disease recurrence is a substantial issue in 1–3 node group despite early stage, PMRT has an instrumental effect in improving LRFS and OS.

## Highlights

• Disease recurrence is a significant concern in post mastectomy patients with T1-T2 N1 disease.
• Post mastectomy radiotherapy (PMRT) has a substantial effect in reducing disease recurrence in this subset of patients.
• PMRT improves disease free survival rate (DFS) and overall survival rate (OS).

## 1 Introduction

Since the Halstedian era of Radical mastectomy to breast conservation and radiotherapy the indications and techniques have been evolving continuously to provide effective local control. Evidence has already proven the benefits of local radiotherapy after mastectomy in 4 or more positive nodes by providing substantial control of any residual microscopic disease [1]. The Early Breast Cancer Trialists Collaborative Group (EBCTCG) published the review of all trials addressing loco regional treatment and accepted the fact that adjuvant loco regional radiotherapy improves loco regional control with impact on long term survival. It is an established fact that one of out every 4 local recurrences developed distant metastasis and over a period of 15 years had a negative impact on overall survival while the outcome in 4 or more positive nodes is known; uncertainty exists in patients with 1–3 positive nodes [2]. Substantial amount of data has been published to address the controversial role of radiotherapy in the management of post-mastectomy patients with 1–3 positive nodes [3,4]. The St. Galen Consensus Conference 2019 on early breast cancer treatment standards with consensus post-mastectomy radiotherapy in N+ 1–3 with adverse features such as TNBC was recommended (yes 85% vs no 8%) [5]. The ASTRO, ASCO, SSO panel without dissent agreed that the available evidence shows that post mastectomy radiotherapy (PMRT) reduces the risks of loco regional failure, any recurrence and breast cancer mortality for patients in this specific category [6]. The long-term analyses of the Danish and British Columbia trials suggest that radiation should be considered as a part of standard treatment in 1–3 node disease [7]. Extrapolating data of MA 20.0 trial in mastectomy setting would have been a logical argument that PMRT is of additional advantage in node positive patients to reduce LRR [8].

Several retrospective studies published in the last era advocated the role of radiation therapy in 1–3 nodes axillary nodal positivity when other adverse prognostic factors i.e. younger age, high histologic grade, presence of lymphovascular invasion (LVI), extracapsular extension of tumour deposit (ECE) and/or oestrogen receptor (ER), progesterone receptor (PR) negative status were present making the case stronger in 1–3 nodes situation [3,4,[9], [10], [11]].

One of the main goal of this retrospective cohort study was to predict the incremental benefits of PMRT in reducing loco regional recurrences and distant metastasis thus improving disease free survival and overall survival in intermediate risk category (1–3 nodes) of breast cancer patients having smaller tumour size but aggressive biology and tumour characteristics that might play a dominant role than number of lymph nodes in specifying comprehensive radiotherapy decision. Local data is not available this study will provide valuable input to our practices.

## 2 Materials and methods

A retrospective cohort study was conducted in the Section of Breast Surgery at two Cancer Centres (Aga Khan University Hospital and Liaquat National Hospital) of Pakistan. The study was registered under the German Clinical Trials Register (DRKS) in accordance with the declaration of Helsinki (Registration ID: DRKS00024402, https://www.drks.de/drks_web/navigate.do?navigationId=trial.HTML&TRIAL_ID=DRKS00024402) and was conducted according to the STROCSS statement guidelines 2019 [12]. The clinical and pathological data of breast cancer cases who were treated at these facilities were collected from medical records from 1998 to 2018. The inclusion criteria were 1) all Breast Cancer patients who had Modified Radical Mastectomy with pathology result showing T1-T2 &1–3 positive nodes who did or did not receive PMRT. 2) Pathology report confirming negative margins. 3) Complete record of oestrogen receptor (ER), progesterone receptor (PR) and human epithelial growth factor receptor (HER-2) status. 4) Number of excised nodes after axillary dissection in double digits i.e. ≥ 10 nodes. Those patients who received Neoadjuvant treatment, diagnosed as Stage IV after initial surgery, having incomplete medical records or lost to follow-up were excluded from the study.

Ethical exemption was obtained from Aga Khan University's and Liaquat National Hospital's Ethical Review Committee (reference number: 2019-1409-3555).

### 2.1 Clinical & pathological data

Patient's clinical and pathological data including age at diagnosis, menopausal status, pT size, type of tumour, number of positive lymph nodes, lymphovascular invasion, margin status, immunohistochemical (IHC) status of oestrogen, progesterone and Her2 neu status, grade of tumour, extra capsular extension of tumour deposit and PMRT data, type of systemic chemotherapy, hormonal therapy, immunotherapy, date of recurrence, type of recurrence (Local, Regional or both) and survival status were collected.

Post mastectomy Radiotherapy technique was standard, the total radiation dose was 50Gy with 2Gy delivered over 25 days over 5 weeks. The chest wall was treated with 6 MV X-rays with opposed tangential fields with the use of tissue equivalent material bolus of 0.5–1 cm when required. Single field irradiation was given for supra and infra clavicular lymph nodal drainage basins with 6 MV X–Ray.

### 2.2 Follow-up and survival endpoints

The patients visited clinic once every 6 months for two years after initial diagnosis and then on annual basis. Thorough history and examination performed at every visit to assess clinically for any signs & symptoms of disease recurrence both local and distant. If required patients were also subjected to radiological imaging including Ultrasound, CT scan or a PET/CT scan.

Loco‐regional recurrence was defined as tumour recurrence at chest wall, ipsilateral axilla, ipsilateral supraclavicular fossa, ipsilateral internal mammary or ipsilateral infra clavicular nodes while distant recurrence was defined as recurrence at any other site. All loco regional recurrences were subjected to histopathological diagnosis with immunohistochemical testing for ER/PR and Her2 neu.

Distant metastasis was diagnosed with CT scan or Bone scan or PET scan and if required histologic proof was also obtained.

Outcome or survival was assessed thru three parameters including overall survival (OS), loco regional free survival (LRFS) and disease-free survival (DFS). Overall survival is defined as survival from the date of diagnosis until death for any reason or the date of last follow up. Loco regional free survival (LRFS) is defined as survival from date of diagnosis till loco regional recurrence developed. Disease free survival is defined as survival from the date of diagnosis until disease recurrence or death.

### 2.3 Statistical analysis

Data was entered and analysed on statistical package for the social sciences (SPSS) (version 23.0). The categorical variables was represented by frequencies and percentages and they were assessed by chi-square/fishers exact test. The quantitative variables were reported as means and standard deviation/median (IQR) and assessed by independent-t-test/Mann Whitney test where appropriate. The variables with p value less than 0.05 by univariate analysis were considered for multivariate analysis. P value of less than 0.05 was taken as significant.

Data was analysed retrospectively to evaluate all potential prognostic factors which were reported previously in multiple studies advocated the role of XRT in 1–3 nodes in the presence of one or more adverse prognostic factors i.e. age, high histologic grade, presence of LVI, extracapsular extension of tumour deposit, molecular subtype and/or ER/PR negative status.

## 3 Results

Patient's data are summarized in (Table 1). A total of 1620 patient files were reviewed out of which 755 qualified the inclusion criteria. Among these cohort, more than half of them 464(61.5%) did not receive PMRT while 291(38.5%) received it. A comparison of patient's clinical and pathological data between these two groups was carried out and evaluated by applying Pearson's chi-squared test taking p-value of less than 0.05 as significant. The analysis identified that mean age of patient in PMRT vs Non PMRT was 52 years vs 53 years respectively and median number of excised lymph nodes were 16 (range: 01–53).

**Table 1:** Demographic and clinicopathological data.

Variable	Total n = 755	Without PMRT n = 464 (61.5%)	With PMRT n = 291 (38.5%)	p-value
Age (year)				
<35	155	86 (18.5)	69 (23.7)	0.096
>35	600	378 (81.5)	222 (76.3)	
Pathology				
IDC(Infiltrating Ductal)	687	422 (90.9)	265 (91.1)	0.003
Non-IDC	68	42 (9.1)	26 (8.9)	
T classification				
1 (2 cm or less)	86	61(13.1)	25 (8.6)	0.060
2 (2–5 cm)	669	403 (86.9)	266 (91.4	
Lymphatic/Vascular Invasion(missing n 38)				
Negative	575	373 (64.9)	202 (35.1)	
Positive	142	60 (42.3)	82 (57.7)	<0.001
Histologic Grade				
1–2	496	330 (71.1)	166 (57)	<0.001
3	259	134 (28.9)	125 (43)	
Oestrogen/Progesterone Receptor(ER/PR)				
ER/PR Positive	556	197 (77.4)	197 (67.7)	
ER/PR Negative	199	105 (22.6)	94 (32.3)	<0.002
Molecular Subtype (missing n 196)				
Luminal A	326	197 (61.4)	130 (54.6)	0.084
Luminal B	67	38 (11.8)	29 (12.2)	0.058
Triple Negative	106	56 (17.4)	50 (21.0)	<0.002
Her 2 enriched	59	30 (9.3)	29 (12.2)	0.037
Positive Lymph Node				
1	386	253 (54.5)	133 (45.7)	
2–3	369	211 (45.5)	158 (54.3)	<0.001
Extracapsular Extension				
Positive	223	87 (18.8)	136 (46.7)	<0.001
Negative	532	377 (81.3)	155 (53.3)	
Chemotherapy				
Yes	675	412(88.7)	263(90.4)	
No	80	52(11.3)	28(9.6)	

Abbreviations: PMRT, Post Mastectomy Radiotherapy Therapy; HER2, human epidermal growth factor receptor 2; Molecular subtype (Missing values are numbers before 2001).

### 3.1 Characteristics of patients in PMRT cohort

The number of patients presented with Luminal A 130 (54.6%), Luminal B 29(12.2%), Her 2 enriched 29 (12.2%) and TNBC breast cancer were 50(21%) respectively. Extra capsular extension seen in 136 (46.7%). Molecular subtyping was missing in 196 cases as Her 2 neu testing was not established as a standard before 2001. Adjuvant chemotherapy was received by 89.4% of cases, CMF was received by only 12.6%, FAC used in 22.5%, FEC in 1.2% cases. Anthracycline and taxane based regimens was given to the majority and trastuzumab was received by 27 patients, hormonal therapy given to 74.5% while 26.4% did not receive hormonal treatment. PMRT group (90%) received chemotherapy vs 88% in non PMRT group.

### 3.2 Prognostic indicators

Factors associated with poor prognosis such as lymphovascular invasion (p < 0.001), high nuclear grade (p < 0.001), 2–3 number of lymph nodes involvement (p < 0.001), ER/PR negative (<0.002), triple negative (p < 0.002) and presence of ECE (p < 0.001) were more likely to undergo PMRT group as compared to non-PMRT group.

On univariate analysis, high nuclear grade (p-0.001), ER/PR negative (0.001), triple negative (p- <0.002), Her 2 neu (p-<0.037) presence of lymphovascular invasion (p-0.001), presence of extra capsular extension (p-0.001) and no radiotherapy (p-0.053) were associated with a significantly higher rate of Loco regional recurrences (LRR). Accordingly, ER/PR negative (HR = 1.1), triple negative (HR = 1.25), Her 2 neu (HR = 1.8) presence of lymphovascular invasion (HR = 1.3), presence of extra capsular extension (HR = 1.4) and age less than 35 years (HR = 1.7), 2–3 nodes positive (HR = 1.6) were the risk factors for Loco regional recurrences in cases who did not receive radiation with statistical significance in the multivariate analysis (Table 2).

**Table 2:** Multivariate analysis for risk factors for loco-regional recurrence in patients.

Without PMRT			With PMRT	
Characteristic	**Hazard ratio (95%Confidence Interval)**	**p-value**	**Hazard ratio (95%Confidence Interval)**	**p-value**
Age (years)				
<35	1.723 (.936–3.169)	<0.002	.257 (.174–.340)	<0.001
>35				
ER/PR status	1.109 (.772–1.595)	<0.002	117 (.048–.186)	<0.002
Negative				
Positive				
Lymphatic/Vascular Invasion Positive	1.121 (.864–1.453)	<0.001	20 (.018–2.32)	<0.003
Negative				
Extra capsular extension	1.488 (.807–2.744)	<0.001	70 (.154–3.16)	<0.001
Positive				
Negative				
Number of Positive Lymph Nodes 2–3 vs 1	1.607 (.260–9.922)	<0.001	1.048 (.661–1.660)	<0.001
Molecular Subtype				
Luminal A vs Triple Negative	1.25 (.186–5.661)	<0.001	.025 (.000–29.463)	<0.001
Luminal A vs Her 2 neu	1.814 (.562–5.852)	<0.002	.030 (.000–8.371)	<0.001

Abbreviations: PMRT, Post Mastectomy Radiotherapy Therapy; HER2, human epidermal growth factor receptor 2.

### 3.3 Survival

During the median follow-up of 78 months (minimum 24 months and maximum 336 months), marked difference was noticed as great majority of patients 74 (24.4%) in non PMRT suffered from Loco regional recurrence than PMRT patients 4 (4.5%) with p < 0.000. Similarly, 24 patients of non-PMRT group suffered from distant metastasis as compared to only 01 in PMRT group giving a significant p-value of less than 0.000.

**Fig. 1:**
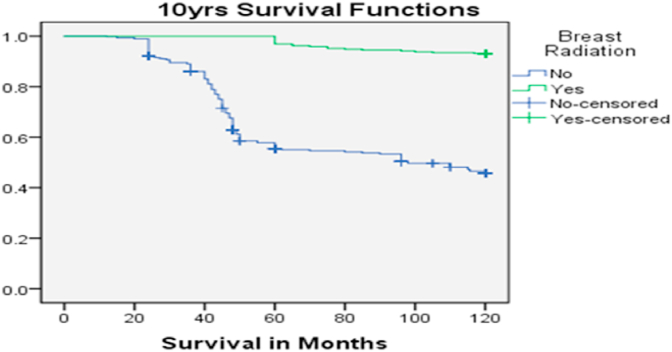
Over Survival with and without Post mastectomy radiotherapy.

**Fig. 2:**
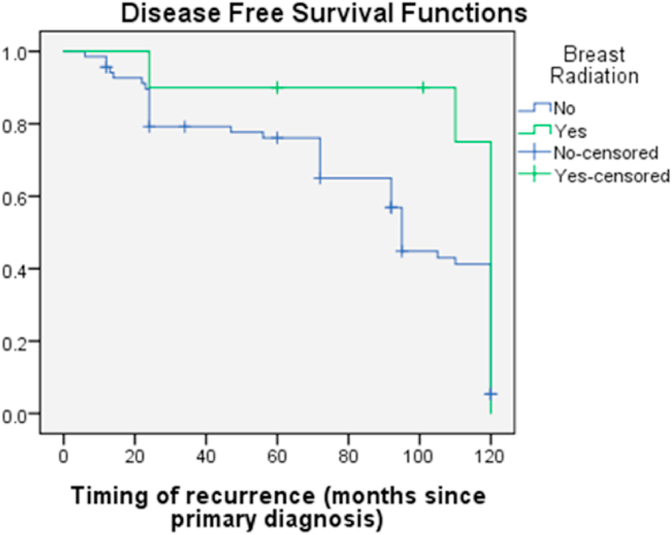
Disease Free Survival with and without Post mastectomy radiotherapy.

A large number of non-PMRT patients eventually succumbed to death (42% vs 8.2%, p-value<0.001). Therefore, the disease-free survival rate and overall survival rate was significantly better for PMRT patients than non-PMRT patients 9.0yrs.vs 5.0 yrs at 10 year follow-up (p = 0.000) and 9.6 yrs vs 4.8 yrs giving a significant p-value<0.000) (Fig. 1, Fig. 2).

**Table:** 

	5 Years		10 Years		15 Years		20 Years	
	with	Without	with	without	with	Without	with	Without
Median	4.9175	3.9978	9.6220	4.8168	14.2818	5.1034	18.836	18.836

**Table:** 

	5 Years		10 Years		15 Years		20 Years	
	with	Without	With	without	with	Without	with	without
Median	5.0000	2.0000	9.0000	5.0000	9.0000	6.0000	9.0000	6.0000

For independent risk factors the disease free survival for PMRT vs Non PMRT group were: age 89.9% vs 70.9% (0.003), triple negative 88.3 vs 71.4%(0.003), LVI 89.6% vs 43%, extra capsular extension 86.8 vs 59.8 (0.000), 2–3 nodes 89.2 vs 73.5%(0.00) respectively.

PMRT improved LRFS & DMFS (p = 0.000) hence DFS 94.3% vs 67.7% (p = 0.000). For those who developed distant metastasis either they were de novo or synchronously with local recurrence in non PMRT group hence data validate the fact that PMRT improved not only LRFS but also the DMFS.

## 4 Discussion

This study is a first large series from Pakistan evaluating the efficacy of PMRT in 1–3 positive node group who had Modified Radical Mastectomy with adequate axillary clearance. Our study supports the hypotheses that benefit of radiotherapy is quite distinct in group with 1–3 positive nodes who received radiation post mastectomy than in non PMRT group. This study could act as a benchmark for developing local guidelines to subject high risk patients to post mastectomy radiation in early breast cancer.

The patients were subjected to radiotherapy based on prognostic factors (age, high histologic grade, presence of LVI, extracapsular extension of tumour deposit, molecular subtype and/or ER/PR negative status) at the discretion of multidisciplinary tumour board meeting. The results showed that radiotherapy has improved the loco regional control and distant recurrences in PMRT vs Non PMRT. At median follow up of 78 months the disease-free survival rate and overall survival rate was significantly superior for PMRT patients (p < 0.000).

Our study identified that Loco regional recurrence (LRR) is an important predictor of DFS. Interestingly we found that only 4 (1.4%) patients developed loco regional recurrences in radiotherapy versus 74(15.9%) in the no radiotherapy group and the distant recurrence was seen in 34 cases of non PMRT group. Our results support other retrospective studies that the lack of PMRT is a strong predictor of a shorter DFS which they correlated with distant metastasis and greater lymph node involvement in triple negative subtype and/or high nuclear grade [[9], [10], [11]].

We reviewed Regional and international data to identify factors associated with improved DFS and OS. Cosar et al. showed that LRR was significantly higher in non PMRT vs PMRT (17% vs 3%) with noteworthy improvement in DFS (p 0.034), similar findings were reported from China reported LRR rate 1.1% and 90.1% in PMRT vs Non PMRT respectively at median follow up of 65 months. Both of these study results did not show any improvement in the overall survival the reason could be a small sample size and short follow up period [3,9]. Recently, results from the Breast International Group 02–98 trial also showed no significant improvement in OS in two cohorts with or without RT which they postulated that use of modern systemic chemotherapy obviates the value of PMRT in improving OS. Nevertheless, majority of our patients received anthracycline and taxane based chemotherapy and where needed trastuzumab was given in both the cohorts [13]. Overgaard et al. have performed subgroup analysis of the DBCG 82 b&c randomized trials to evaluate the loco-regional recurrence rate and survival in relation to number of 1–3 positive nodes, they limited the analysis to 1152 node positive patients with 8 or more nodes removed. They reported improved overall 15-year survival rate in this subgroup 39% and 29% (p = 0.015) with or without radiation which is consistent with our results [14]. Meta-analysis on role of PMRT in 1–3 nodes regardless of use systemic therapy identified that the majority of retrospective studies used had shorter follow up time (53–150 months), they indicated that longer follow-up time may allow the significant benefit of reducing LRR and distant recurrence to translate to an increased benefit in OS which is a fair explanation as in our study the follow up time was significantly longer as reported in meta-analysis also (24 months–336 months) [11].

The logical question is whether radiotherapy is equally effective in situations where there is small tumour size with comparatively smaller disease burden i.e. 1-3 positive nodes? Another pertinent question cam be asked, is it logical to determine XRT need based on the number of positive nodes only? Overgaard and his group have reached the conclusion in their sub group analysis that taking the decision to irradiate only on the basis of number of lymph nodes is a crude method to define the potential need to recommend radiation, more strong recommendation can be made on the basis of lymphovascular invasion, extra nodal extension of tumour, molecular subtype, young age and high grade in intermediate risk category i.e. 1-3 nodes positive [14].

Multivariate analysis of our cohorts has proved that low burden disease i.e. one node positive in the presence of other poor prognostic factors did not show any significant benefit in improving LRR or OS. This is consistent with results from several other retrospective reviews and trials. However, 2–3 node positive disease with molecular subtype and/or ER/PR negative, presence of LVI, high nuclear grade, extra capsular extension of tumour deposit reached statistics significance in improving LRR, DFS, DMFS and OS.

Several theories have been proposed to justify the potential benefit of radiotherapy (RT) in TNBC. Accepting the fact that ER/PR – or triple negative are a poor prognostic group with highly proliferative, poorly differentiated, high grade disease aggressive nature. It is frequently associated with a BRCA-1 mutated pathologic subtype; its presence can reduce the capacity of DNA repair which enhanced the radio sensitivity of TNBC cells [15]. Moran hypothesized that different biological subtypes within TNBC have different radio sensitivities that could be the reason that despite lack of level 1 evidence these cohort do better with radiation [16]. Another potential mechanism could be that radiation promotes cancer cell autophagy which is recognized as having the potential to contribute to cell killing in response to a variety of chemotherapeutic agents as well as ionizing radiation [17]. However conflicting reports has been published from separate studies on impact of PMRT in different molecular subtypes of breast cancer in reducing LRR and improving survival. Contrary to our finding a retrospective analysis of 16521 from SEER data showed PMRT significantly prolonged survival in Luminal A patients, their results were consistent with findings from Danish Breast Cancer Cooperative group [18,19]. Congruent to our results retrospective analysis of 1369 patients from China reported PMRT reduced LRR rate in TNBC, but showed no effect on OS irrespective of subtypes. Wang et al. reported that the combination of chemotherapy and radiotherapy could significantly increase five-year recurrence-free survival and overall survival in TNBC women after mastectomy in stage I-II [10,20]. St. Galen Consensus Conference 2019 on early breast cancer treatment standards strongly recommended post-mastectomy radiotherapy in N+ 1–3 with adverse features such as TNBC (yes 85% vs no 8%) [5].

Several retrospective analyses reported association between LRR and survival with LVI, high grade disease or ECE of tumour in 1–3 node positive group [3,9,20]. We observed in our results that grade III disease, presence of LVI and extra capsular extension is associated with improved prognosis and lesser recurrences.

A retrospective analysis from Cleveland clinic has shown that LRR rate was 50.4% in non-radiation group with both Grade III disease and ECE over 5 years, similarly Katz et al. reported that high grade disease with ECE >2 mm experienced high rate of LRR without RT [21,22]. Evidence has suggested that LVI is an adverse prognostic factor for relapse and survival particularly in triple negative breast cancer [23,24]. Based on their analysis Ahn et al. concluded that adjuvant RT minimized the negative prognostic effect of LVI on DFS p = 0.068 [with RT] vs. p = 0.011 [without RT] [25]. Cosar at al associated LVI with improved OS [3].

Another compelling fact the study revealed is that the Her 2 neu enriched tumours also demonstrated marked difference in LRFS between two groups however half of the studied patients did not receive Trastuzumab hence we could not ascertain with confidence that Her 2 neu molecular subtype may benefit from radiotherapy in 1–3 node positive group the same observation has been shared by Zhen et al. [3]. Due to the fact that patients who received trastuzumab have a very low rate of LRR as compare to other molecular subtypes with that in mind it is inexplicit whether targeted treatment may modify the study result [26].

There are certain limitations to this study. First, inherent selection bias exists as this is a retrospective chart review therefore there might be some missing data and information that was included besides small sample size. Second, multivariate model was used to control confounders however there is a possibility of unmeasured confounders during data collection.

Patient were assigned to radiotherapy based on decision of multidisciplinary tumour board, the decision was taken based on available evidence from meta-analysis and well-designed controlled trials.

The only solution to uniformly address this grey zone is RCT hence clinicians are desperately waiting for result of 02–04 MRC EORTC SUPREMO trial which recruited >1600 patients prospectively between April 2007 and May 2013. This trial aim to determine the effect on overall survival of chest wall irradiation after mastectomy and axillary surgery in women with operable breast cancer at ‘intermediate-risk’ of loco regional recurrence and who also received modern systemic therapy [27]. However, the results of this trial are expected in 2023 at the earliest, hoping much of the controversy will be resolved with their data!

## 5 Conclusions

Disease recurrence is a substantial issue in 1–3 node group despite of early stage, crux of all treatment efforts in adjuvant setting is to avoid recurrence. We hypothesized that radiotherapy is acting against microscopic residual disease post mastectomy resulting in reduced LRR and improved Survival.

## Availability of data and materials

The datasets used and/or analysed during the current study are available from the corresponding author on reasonable request.

## Ethical approval

Ethical exemption was obtained from Aga Khan University's and Liaquat National Hospital's Ethical Review Committee (reference number: 2019-1409-3555) which waived documentation of informed consent due to the observational nature of the study.

## Funding resources

The authors have no funding resources to be declared.

## Author contribution

Please specify the contribution of each author to the paper, e.g. study concept or design, data collection, data analysis or interpretation, writing the paper, others, who have contributed in other ways should be listed as contributors.

Lubna M. Vohra: Study Conception, Data Collection, Analysis, Investigation, Writing, Critical review and revision, Final approval of the article, Rufina Soomro: Study Conception, Data Collection, Critical review and revision, Final approval of the article, Dua Jabeen: Analysis, Investigation, Writing, Critical review and revision, Final approval of the article, Nasir Ali: Investigation, Critical review and revision, Final approval of the article, Nargis Khan: Data Collection, Analysis, Critical review and revision, Final approval of the article.

## Provenance and peer review

Not commissioned, externally peer-reviewed.

## Declaration of competing interest

The authors have no conflicts of interest to declare.
